# Schizophrenia and Depression Co-Morbidity: What We have Learned from Animal Models

**DOI:** 10.3389/fpsyt.2015.00013

**Published:** 2015-02-18

**Authors:** James N. Samsom, Albert H. C. Wong

**Affiliations:** ^1^Department of Molecular Neuroscience, Centre for Addiction and Mental Health, Campbell Family Mental Health Research Institute, Toronto, ON, Canada; ^2^Department of Pharmacology, Faculty of Medicine, University of Toronto, Toronto, ON, Canada; ^3^Department of Psychiatry, Faculty of Medicine, University of Toronto, Toronto, ON, Canada

**Keywords:** mouse, schizophrenia, depression, animal model, genetics

## Abstract

Patients with schizophrenia are at an increased risk for the development of depression. Overlap in the symptoms and genetic risk factors between the two disorders suggests a common etiological mechanism may underlie the presentation of comorbid depression in schizophrenia. Understanding these shared mechanisms will be important in informing the development of new treatments. Rodent models are powerful tools for understanding gene function as it relates to behavior. Examining rodent models relevant to both schizophrenia and depression reveals a number of common mechanisms. Current models which demonstrate endophenotypes of both schizophrenia and depression are reviewed here, including models of CUB and SUSHI multiple domains 1, PDZ and LIM domain 5, glutamate Delta 1 receptor, diabetic db/db mice, neuropeptide Y, disrupted in schizophrenia 1, and its interacting partners, reelin, maternal immune activation, and social isolation. Neurotransmission, brain connectivity, the immune system, the environment, and metabolism emerge as potential common mechanisms linking these models and potentially explaining comorbid depression in schizophrenia.

## Introduction

Schizophrenia and depression are devastating mental illnesses that contribute substantially to the global burden of disease (1–3). Moreover, schizophrenia patients have an elevated risk for developing depressive symptoms compared to the already high lifetime prevalence of depression in the general population (4). Depression has been reported during all stages of the course of schizophrenia (5–8), and depressive symptoms are associated with an increased risk of suicide (9, 10). Methodological differences in diagnosis and time course of evaluation mean that there is a wide variance of depressive symptoms reported by patients with schizophrenia in the literature, with prevalence rates as high as 61% (11). Nevertheless, reviews of the literature convincingly show that depression is elevated in schizophrenia (4).

Conversely, depressed patients have also been shown to be at a higher risk of developing psychosis, and depression is often seen in people at high risk for schizophrenia prior to the emergence of psychotic symptoms (12–17). Furthermore, the emergence of psychotic symptoms in depression, considered as a distinct clinical subtype of depression called psychotic depression or depression with psychotic features, is associated with increased severity of depressive symptoms (18, 19). This mutual relationship of risk between schizophrenia and depression suggests potential overlap in the pathophysiology and/or etiology of the two disorders.

The relationship between psychotic and affective symptoms has been a controversial issue within psychiatric nosology for years. A central question in the debate is reflected by the discussion surrounding schizoaffective disorder, which currently remains a distinct diagnosis characterized by the presence of a major mood episode (depressive or manic) concurrent with schizophrenia (20). Low diagnostic reliability has led some to question the inclusion of schizoaffective disorder as a separate condition (21, 22). It remains unclear whether depressive symptoms should be considered as a symptom of schizophrenia, comorbid symptoms, or unrelated epiphenomena (23).

There is an overlap between certain negative symptoms of schizophrenia and depressive symptoms; for example, anhedonia, abulia, alogia, amotivational and avolitional states, and social withdrawal (24). Hence, some argue depressive symptoms should be part of the schizophrenia syndrome (25–28). This view is supported by the high prevalence of depressive symptoms in schizophrenia and the association between trait depression and other trait-like features of schizophrenia. Alternatively, depressive symptoms in schizophrenia could partly be a side-effect of neuroleptics, secondary to other comorbidities such as substance abuse, or an understandable reaction to the consequences of the disorder (29–34). Regardless of the status of depressive symptoms as core or comorbid with schizophrenia, there is clearly some overlap in the presentation of both disorders.

There is increasing evidence of shared genetic risk factors for both schizophrenia and depression. A genome wide association study (GWAS) of five major psychiatric disorders found that SNPs within chromosomal regions 3p21 and 10q24, and calcium channel subunit genes *CACNA1C* and *CACNB2* were significantly associated with schizophrenia, depression, bipolar, attention deficit-hyperactivity (ADHD), and autism spectrum disorders (ASD) (35). Additionally, the subgroup of schizophrenia patients who also suffer from depression has proved useful in finding genetic associations. The NMDA receptor subunit gene *GRIN1*, the hippocampal stress modulating glycoprotein gene *GPM6A*, and chromosomal regions 4q28.3 and 20q11.21 were associated with depression comorbidity in schizophrenia patients (36–38). These studies hint at potential shared molecular pathways that underlie both schizophrenia and depression.

The etiology and pathophysiology of both schizophrenia and depression remain poorly understood. It is clear that there is some relationship between the two disorders, which affects the risk and severity of disability for both disorders. Understanding the neurobiology linking schizophrenia and depression could provide great insight into both disorders. Animal models are some of our best tools for understanding the complex pathways that connect genes and behavior. Many excellent reviews have been written on the use of animal models in studying neuropsychiatric disorders and outlining many of the current models (39–42). Rather than reviewing the literature on neuropsychiatric disorders as a whole or focusing specifically on one disorder, this review will focus on what animal models can teach us about schizophrenia and depression comorbidity. Here, we provide a broad overview of the rodent models that express phenotypes resembling comorbid schizophrenia and depression, and what they reveal about the neurobiology of comorbidity in psychiatric illness.

## Finding Animal Models for Neuropsychiatric Disorders

When modeling neuropsychiatric disorders in animals, it is desirable that the criteria for the three types of validity are fulfilled: face (i.e., similar symptoms), construct (i.e., similar etiology or genetic/environmental cause), and predictive (i.e., responds to relevant drug treatments) (39, 43). Given that depression and particularly schizophrenia are defined by complex multidimensional sets of symptoms that can be highly heterogeneous between patients, it has been proposed that these disorders may be approached by examining endophenotypes, which are easier to measure, and may be more proximal to the underlying genetic and biological mechanisms (44). Therefore, the typical approach for modeling these disorders in mice or rats is to manipulate some genetic or environmental factor, which has a plausible etiological link to either schizophrenia or depression, and then examine the animal for endophenotypes that resemble those seen in either disorder. Models typically will display only a subset of all the endophenotypes, which define either disorder, which is expected given the heterogeneous and polygenetic nature of both schizophrenia and depression.

Behavioral endophenotypes have been particularly useful for studying neuropsychiatric illness in rodent models. For instance, certain features of schizophrenia have behavioral correlates that are measurable in rodents. Pre-pulse inhibition (PPI) is a phenomenon in which the response to a stimulus is inhibited by a preceding similar stimulus. For example, the startle response to a loud noise is less intense if a quieter preceding warning noise is played. PPI deficits are seen in schizophrenia patients and their unaffected relatives, and are measurable in rodents (45–48). PPI is framed as a measure of sensorimotor gating, which is known to be affected in schizophrenia (49). Sensorimotor gating deficits in rodents can also be exhibited as sustained hyperactivity in a novel environment caused by a failure to habituate to novel stimuli (50).

Cognition is another area in which many sophisticated rodent tests have been developed. Even complex cognitive processes such as executive function are measurable in rodents. For instance, a rat version of the Wisconsin-card-sorting test used to measure the ability learn rules and adapt to change in humans has been developed (51). Rats must switch between learned scent and texture cues to locate hidden food rewards. Schizophrenia patients are known to have deficits in executive function, and have impaired performance in the Wisconsin-card-sorting test (52–55). Animal behavioral tests that can predict clinical drug effects in humans are also important. For example, antidepressants can reduce immobility in the forced swim test (FST) and tail suspension test (TST). Therefore, these behavioral tests have been used to screen potential antidepressant medications (56, 57). Increased immobility in these tests has been suggested to indicate behavioral despair or the inability to cope with stress, but the meaning of these tests and how they translate to behavior in humans remains unclear (39). Table 1 provides a few examples of rodent behavioral paradigms that are relevant for neuropsychiatric disorders.

**Table 1:** **Mouse behavioral phenotypes related to neuropsychiatric disorders**.

Mouse behavioral test	Ethological correlate	Disease associations
Elevated plus/0 maze	Decreased time in open arms of a maze with open and enclosed arms models state anxiety (58)	BPD, GAD, OCD, panic disorder, phobias, PTSD (59)
Forced swim/tail suspension test	Increased immobility possibly related to behavioral despair or coping with stress (55)	Related to antidepressant activity, depression (39)
Latent inhibition	The effectiveness of conditioning in mice previously exposed vs. not exposed to a stimulus. Related to the ability to ignore irrelevant stimuli during learning (60)	SCZ (61)
Morris water maze (MWM)	Latency to find a hidden platform in a pool of murky water and memory for platform position tests spatial learning and memory (62)	AD, OCD, SCZ (63–65)
MWM reversal learning	Ability to learn a new position when the platform is moved from its previous position tests cognitive flexibility (66)	ADHD, ASD, Huntington’s, OCD, SCZ (67–69)
Novel object recognition test	Preference for exploring new vs. familiar objects tests recognition memory, episodic memory, and visual attention (70, 71)	ADHD, ASD, learning disability, PTSD, SCZ (72–74)
Open field test	Tests exploration and motor activity (75)	Altered motor activity linked to ADHD, BPD, depression, SCZ (53, 76)
	Willingness to enter the center of the field measures anxiety (75)	BPD, GAD, OCD, panic disorder, phobias, PTSD (59)
Pre-pulse inhibition	Magnitude of the startle response to a loud noise in the presence and absence of a preceding noise, tests sensorimotor gating (74)	ASD, Huntington’s, OCT, SCZ, Tourette’s (77)
Psychostimulant-induced locomotor activity	Excess hyperactivity after injection with a psychostimulant tests sensitivity. Connected to the functioning of brain reward circuits (78)	Drug addiction, psychostimulant-induced mania, SCZ (39, 79, 80)
Set-shifting test	Ability to switch between different cues to locate a food reward tests cognitive flexibility (54)	ADHD, ASD, Huntington’s, OCD, SCZ (68, 69)
Sucrose preference test	Mouse preference for sugar vs. normal water tests anhedonia (81)	Alcohol dependence, depression, hysteria (82, 83)
T-maze/Y-maze	Alternation of entry onto the arms of the maze during reward retrieval (T-maze)/exploration (Y-maze) tests working memory (84, 85)	SCZ (68)
Three-chamber social interaction test	Time spent with mouse vs. object tests social motivation, time spent with familiar vs. new mouse tests social memory (86)	ASD, BPD, depression, SCZ (87–91)

*AD, Alzheimer’s disease; ASD, autism spectrum disorder; ADHD, attention deficit-hyperactivity disorder; BPD, bipolar disorder; GAD, generalized anxiety disorder; OCD, obsessive–compulsive disorder; PTSD, posttraumatic stress disorder*.

Disorders of the human brain are complex, and while animal models are useful tools in understanding neurobiology and gene function, caution must be exercised when using animal models for the translational study of human psychiatric disorders. Despite high conservation of gene structure and function, there can be large interspecies differences in gene expression patterns, gene regulation, and protein translation between human and mouse or rat. Furthermore, the correlations between genes, biology, and behavior we measure in animals may not map perfectly to the symptoms in humans we wish to model. Behavioral findings are sometimes idiosyncratic and specific to particular laboratories (92), so it is sometimes difficult for models developed in one lab to be replicated and used by others without careful attention to environmental and test conditions (93).

Nevertheless, animal models have greatly contributed to the understanding and treatment of neuropsychiatric disorders. For instance, latent inhibition (LI) is a class of cognitive tests that measure a learning effect in which exposure to conditioned stimuli alone in associative learning paradigms can interfere with subsequent learning. LI is an established model of attentional deficits in schizophrenia (61). Assessing mouse LI has been productive in validating new candidate genes for schizophrenia (94). Additionally, animal behavioral testing has been integral in assessing the efficacy of new compounds with potential antidepressant and antipsychotic effects (95–97).

## Models

Many animal models have been generated to explore various factors associated with both depression and schizophrenia. The presence of both schizophrenia and depression-related endophenotypes in a single model may be useful in understanding comorbidity and the shared symptomology between the two disorders. Hence, this review will focus exclusively on rodent models that display endophenotypes relevant for both schizophrenia and depression. Investigating the common elements between such models may provide clues about the shared pathways which lead to comorbidity for these two disorders.

### CUB and SUSHI multiple domains 1

Human genetic studies have found significant links between *CSMD1* and schizophrenia, with nominally significant links reported for depression and bipolar disorder (98–102). Furthermore, risk variants in *CSMD1* were shown to have effects on cognition and brain activation in healthy participants (103, 104). CUB and SUSHI multiple domains 1 (CSMD1) is a complement control-related protein that inhibits C3 *in vitro* (105, 106). Complement is tightly regulated in the CNS as it is involved in microglia-dependent synaptic pruning and phagocytosis. For this reason, it is hypothesized that CSMD1 could play a role in aberrant synaptic elimination in neurodegenerative disorders (107). Hence, both immune and synaptic regulation may mediate the effects of Csmd1 in the development of both schizophrenia and depression-related phenotypes.

Steen et al. generated a Csmd1 knockout (KO) by deleting a 1 kb sequence from exon/intron1 (108). These mice developed an anxiety-like phenotype in the open field test (OFT) and elevated plus maze (EPM). The mice also had increased exploratory activity in the novel object recognition test (NORT). However, this did not affect working memory, recognition, or preference, so it may simply be a reflection of increased anxiety. The mice had a potential depression-like phenotype in the TST, but no changes were seen in PPI. Finally, the KO mice had a significant increase in body weight accumulation over time, and increased glucose tolerance.

Interestingly, a previous study tested an exon1 deletion Csmd1-KO mouse on schizophrenia endophenotypes (109). This group found no significant changes in schizophrenia-related behaviors: PPI, social interaction test (SIT), sucrose preference test (SPT), or sensitivity to amphetamine in the amphetamine-induced locomotor activity test (amphetamine-ILAT). This agrees with the results of Steen et al.; however, this group did not test for depressive endophenotypes. The lack of a significant effect on classical schizophrenia endophenotypes may be due to mouse–human differences in CSMD1. Nevertheless, Steen et al. suggest that CSMD1 may play a role in the common symptoms between bipolar disorder, depression, and schizophrenia. Steen et al. found that depletion of Csmd1 had very little effect on the whole transcriptome, and identified a Csmd1 promoter-associated lncRNA, possibly responsible for brain-specific promoter activity in the CNS. This suggests that Csmd1 was directly responsible for the manifestation of schizophrenia and depression-like behavior in these mice (108).

### PDZ and LIM domain 5

PDZ and LIM domain 5 (PDLIM5) has been associated with schizophrenia, depression, and bipolar disorder in human genetic and expression studies (110–117). *PDLIM5* encodes the enigma homolog (ENH), of which five protein isoforms have been identified in humans (118). PDLIM5 is known to interact with protein kinase C (PKC), and may be involved in the regulation of intracellular calcium levels through PKC epsilon (PCKE) and Ca^2+^ channel interactions (119). In the nervous system, PDLIM5 is localized in presynaptic terminals and the postsynaptic density; furthermore, PDLIM5 was shown to interact with spine-associated RapGAP (SPAR, SIPA1L1), and to stimulate the shrinkage of dendritic spines (120, 121). Combined with the genetic results implicating L-type calcium channel genes *CACNA1C* and *CACNB2* in schizophrenia, depression, bipolar disorder, autism, and ADHD, this model further implicates Ca^2+^ channels in neuropsychiatric illness (35). Hence, Pdlim5 may affect schizophrenia and depression-related behaviors through regulation of Ca^2+^ channels as well as synapse regulation.

Horiuchi et al. generated a *Pdlim5* KO using a gene trap embryonic stem cell line (122). Homozygotes for the Pdlim5-KO were embryonic lethal; however, heterozygotes were viable with normal weight and brain size. Pdlim5 deficiency in heterozygotes had a protective effect on schizophrenia-like phenotypes in chronic and acute methamphetamine-induced locomotor hyperactivity in the open field (METH-ILAT) and methamphetamine impairment of PPI. Furthermore, the effects on PPI and METH-ILAT were replicated when Pdlim5 was inhibited with PKCE-TIP in wild type mice. Pdlim5-deficient heterozygotes expressed a depression-like phenotype in the TST that was rescued by the antidepressant imipramine. Pdlim5 expression was shown to increase in the prefrontal cortex (PFC) of mice with chronic methamphetamine injection and in the brains of mice with chronic imipramine injection, but did not change with acute dosing or with injection of the classical antipsychotic haloperidol. This study is limited by the lack of data from complete KO mice; furthermore, PKCE-TIP is not specific to Pdlim5. Nevertheless, the data suggest that increased Plim5 levels may cause schizophrenia-like behavioral phenotypes, whereas decreased Pdlim5 may result in depression.

### Glutamate Delta 1 receptor

Human GWAS has associated *GRID1* with schizophrenia, bipolar disorder, and depression (123–127). Glutamate Delta 1 receptor (GluD1) is a member of the orphan family of delta ionotropic glutamate receptors (iGluRs), and has widespread neuronal expression in adult mice particularly in the forebrain, with diffuse expression in the CNS during development (128–131). While typical iGluR ligands fail to generate current responses in the GluD1 receptor, there is evidence that the NMDA receptor allosteric activator d-serine binds to GluD1 receptors (132). d-serine binding is hypothesized to affect receptor function indirectly; for instance, through alteration of dimer stability. Studies *in vitro* suggest GluD1 may be involved in the induction of presynaptic differentiation and synapse formation (133–136). Synaptic regulation and glutamate signaling mediated by GluD1 may influence the development of schizophrenia and depression-related symptoms.

Yadav et al. used targeted disruption to delete exons 11 and 12 of the GluD1 gene *Grid1* in mice (137). GluD1-KO mice showed hyperactivity in the OFT, decreased anxiety-like behavior in the EPM and marble burying tests, depression-like behavior and anhedonia in the FST and SPT, and increased aggressive behavior. GluD1-KO mice had deficits in social interaction, which could be rescued by treatment with the GluN1 NMDA receptor subunit agonist d-cycloserine. The GluD1-KO mice also had enhanced working memory in the Y-maze and radial arm maze, but they had deficits in reversal learning in the Morris water maze (MWM) with no changes in spatial learning, and deficits in cue and contextual fear conditioning, but no changes in LI (138). The authors found significantly higher expression of GluA1, GluK2 (ionotropic GluR subunits), and PSD95, and a trend for higher expression of GAD67 (inhibitory neuron marker) in the amygdala of GluD1-KO mice. They also found decreased expression of GluA1 and GluA2 in the PFC and hippocampus of the KO mice, as well as decreased GluK2 and GAD67 and elevated GluN2B and PSD95 in the hippocampus. Decreased GluA1 levels could be rescued by d-cycloserine treatment. While this mouse lacks schizophrenia-associated deficits in PPI and LI, violence has been associated with schizophrenia (139, 140); therefore, the hyper-aggression seen in these mice could be relevant to schizophrenia. Furthermore, changes in working memory, reversal learning, and anhedonia could reflect cognitive and negative symptoms of schizophrenia.

Given the known role of GluD1 in synaptic regulation, synaptic deficits are likely to underlie the changes in behavior seen in the GluD1-KO mice. However, the exact nature of the effects of GluD1 on the synapses in these mice remains to be thoroughly explored. The expression data also hints at an inhibitory–excitatory imbalance in the synapses of the KO mice. This type of imbalance has also been seen in other animal models of schizophrenia (141–143). Certainly, alterations in synaptic regulation and function are becoming a common theme among animal models showing schizophrenia and depression-like behaviors.

### Diabetic db/db mice

The leptin receptor-deficient db/db mouse is an established mouse model of metabolic conditions such as diabetes mellitus, obesity, and dyslipidemia. The db/db mice were shown to have impaired spatial learning in the MWM accompanied by deficits in long-term potentiation (LTP) (144). Dinel et al. showed that db/db mice have increased anxiety-like behaviors in the OFT and EPM, and impaired spatial working memory at long stimulus intervals in the Y-maze with no impairments in working or recognition memory in the NORT (145). These behavioral deficits were associated with hippocampal inflammation. These authors found the db/db genotype did not associate with depression-like behavior in the FST and TST. A more recent study reconfirmed previous results of impaired memory and anxiety-like behaviors; however, this group found increased immobility time of both juvenile and adult db/db mice in the FST and impaired PPI of adult, but not juvenile mice, suggesting both schizophrenia-like and depression-like phenotypes (146). The contradictory results of the two studies in the FST may be explained by differences in experimental procedures, as increased immobility in the FST is likely to occur with increasing stress, which could be affected by the order and number of tests in each paper.

An analysis of CNS protein expression in db/db mice found overlap in protein expression in shared pathways in neuropsychiatric disorders; notably, decreased peptide YY, which is seen in drug free cerebrospinal fluid of schizophrenia patients, and inflammatory and Ca^2+^ regulatory molecules, which share pathways with cognitive disorders, depression, Alzheimer’s, and schizophrenia (147). Metabolic conditions such as obesity and diabetes are frequently comorbid with depression and schizophrenia (148–151). Furthermore, there is considerable evidence that antipsychotics and possibly schizophrenia itself may disrupt important metabolic pathways (152). The db/db mouse could be useful for linking schizophrenia and depression to important metabolic pathways, which also increase susceptibility to obesity and diabetes.

### Neuropeptide Y

The 36 amino-acid peptide neuropeptide Y (NPY) is widely distributed in the CNS and recognized to play a role in eating behavior, energy balance, and cardiovascular functions (153, 154). The NPY system has been implicated in schizophrenia by post-mortem human studies, which found decreased NPY in the cortex of schizophrenia and bipolar disorder patients (155, 156). The Y2 receptor is also known to interact with the dopamine (DA) system in humans and rodents, providing a further link to schizophrenia (157, 158). The NPY system has been implicated in depression via its role in modulating stress response, mood, and affective behaviors (159–161). Hence, disruptions in the NPY system could potentially be involved in both schizophrenia and depression, while also linking both disorders to metabolic conditions, appetite changes, and obesity.

Multiple rodent models of the NPY system have been used to investigate the role of NPY in depression and schizophrenia. Stadlbauer et al. administered NPY receptor agonist peptide YY (PYY_3–36_) intraperitoneally in mice (162). This treatment caused deficits in social interaction with no significant increase in anxiety-like behaviors in the EPM. Schizophrenia-like deficits in LI and PPI were also induced by PYY administration. PYY-induced PPI deficits could be reversed by haloperidol, but not the atypical antipsychotic clozapine. Additionally, PYY injection impaired spatial learning in the MWM. Y2 receptor-deficient male (but not female) mice displayed hyperactivity in the OFT, increased social interaction, and moderately improved PPI, suggesting a protective effect against schizophrenia-associated behaviors (163). Y2 deficiency also caused decreased anxiety-like behavior in the EPM and OFT (164). Implicating NPY in depression-like phenotypes, administration of the NPY Y1 receptor agonist NPY (Leu31, Pro34) had anxiolytic and antidepressant effects on cholecystokinin-4 (CCK-4)-induced anxiety-like behavior in the SIT and depression-like behavior in the FST (165). Furthermore, it was found that PYY_3–36_ administration increased the immobility time of olfactory bulbectomized rats in the FST (166). These models are part of a larger body of work implicating the NPY system in stress-related depressive disorders (161). These models suggest that activation of the NPY system via Y2 receptors may cause schizophrenia-like behavior while exerting an antidepressant-like effect.

### Disrupted in schizophrenia 1

A chromosomal translocation intersecting *DISC1* was first found in a Scottish pedigree with a high frequency of severe psychiatric disorders, including schizophrenia, depression, and bipolar disorder (167, 168). Additional genetic associations between *DISC1* and neuropsychiatric illness were found in other populations (169–171). Disrupted in schizophrenia 1 (DISC1) is a scaffolding protein implicated in multiple downstream functions, including embryonic and adult neurogenesis; and neuronal proliferation, differentiation, and migration (169, 172–174). DISC1 interacts with many other proteins involved in synaptic function, neurodevelopment, the cytoskeleton, and centrosomal pathways, some of which are also associated with schizophrenia and depression (e.g., AKT, DPYSL2, GSK-3β, PDE4, CREB, and β-arrestin) (175–181). The distinct pathways by which DISC1 mediates its effects have been intensely studied, and a detailed discussion is beyond the scope of this review. Essentially, Disc1 may affect behavior via its roles in neurodevelopment, synaptic transmission, and synaptic plasticity mediated through multiple downstream interacting partners such as PDE4, Ndel1, GSK-3, and Dixdc1.

The numerous animal models that have been generated to investigate the role of DISC1 in the neurobiology of mental illness have been reviewed in considerable detail elsewhere (174). Cognitive, schizophrenia-like, and depression-like deficits are common in the various models, but not all are present simultaneously in every model. Table 2 provides a summary of the behavioral phenotypes of relevant *Disc1* mouse models. Disruptions in Disc1 caused alterations in neurodevelopment, such as changes in brain structure, abberant formation of cortical layers, reductions in GABAergic interneurons, and altered neuronal morphology, maturation, neurite growth, and axonal targeting (182–190). Disc1 alterations also caused reductions in dopaminergic and hippocampal synaptic transmission, and short-term plasticity but not LTP (185–188). Changes were also seen in the activity of downstream Disc1 interacting partners, notably in the PDE4 family of phosphodiesterases and glycogen synthase kinase 3 (GSK-3) pathways (181, 182, 186). This implicates Disc1 in both neurodevelopment and synaptic transmission through its interactions with multiple downstream pathways.

**Table 2:** **Behavioral phenotypes of Disc1 genetic mouse models**.

Name of mouse line	Behavioral phenotypes			Reference
	SCZ-like	Depression-like	Cognitive	
CaMK-DN-DISC1 tg	Hyperactivity, PPI deficits	↑Immobility in FST	Working memory deficit in Y-maze	(184)
CaMK-DISC1-cc tg at PND 7		↑Immobility in FST; ↓sociability	Working memory deficit	(187)
DN-DISC1 tg	Hyperactivity	↑Aggression	Spatial memory deficit in MWM	(189)
*Pre- and post-natal Tet-off* DN-DISC1 tg		↑Immobility in TST; ↓sociability; ↑aggression		(191)
DISC1 KD (transient *in utero* cortical)	PPI deficits		Impaired long-term but normal short-term operant conditioning; working memory deficit in T-maze	(188)
DISC1tr		↑Immobility in FST/TST	Fear memory deficit	(190)
DISC1-129	PPI deficits		Working and fear memory deficits	(185, 192)
DISC1-Q31L	PPI and LI deficits	↑Immobility in FST; social anhedonia	Working memory deficit in T-maze	(181, 182)
DISC1-L100P	Hyperactivity, PPI, and LI deficits		Working memory deficit in T-maze	(182, 193)
**Gene × environment models**
DN-DISC1 tg × polyl:C at E9	Hyperactivity	↑Immobility in FST; ↑anxiety; ↓ sociability		(194)
DISC1-L100P^+/−^ × polyl:C	PPI and LI deficits	↓Sociability	Spatial operant conditioning deficit	(195)
DN-DISC1-Tg-PrP × social isolation	Hyperactivity; PPI deficit	↑Immobility in FST		(196)

*CaMK-DN-DISC1 tg, transgenic mice expressing dominant-negative C-terminal truncated human DISC1 under control of the α-calmodulin kinase II promoter; CaMK-DISC1-cc tg, transgenic mice expressing C-terminal portion of the human DISC1 under control of the-calmodulin kinase II promoter; DN-DISC1 tg, transgenic mice with inducible expression of dominant-negative C-terminal truncated human DISC1 (hDISC1) limited to forebrain regions, including cerebral cortex, hippocampus, and striatum, using the Tet-off system under the regulation of the CAMKII promoter; DISC1 KD, DISC1 knockdown; DISC1tr, transgenic mice expressing two copies of the truncated human DISC1 encoding the first eight exons using a bacterial artificial chromosome; DISC1-129, 129S6/SvEv inbred mouse strain carries a termination codon at exon 7 of DISC1 gene, which abolishes production of the full-length DISC1 protein; DISC1-Q31L, point mutation in the second exon of DISC1 leading to the substitution of glycine on leucine at 31 amino acid of DISC1 protein; DISC1-L100P, point mutation in the second exon of DISC1 leading to the substitution of leucine on proline at 100 amino acid of DISC1 protein. Table adapted from Lipina and Roder copyright (174), with permission from Elsevier*.

### DISC1 interacting partners

Mouse models for a number of DISC1 interacting partners also display behaviors relevant to both schizophrenia and depression. Mice deficient in fasciculation and elongation protein zeta 1 (Fez1) displayed a schizophrenia-like hypersensitivity to psychostimulants and antidepressant-like reduced immobility in the FST (197). These changes were associated with increased DA transmission in the nucleus accumbens. Mice deficient in the phosphodiesterase PDE4B not only showed a similar behavioral phenotype to the Fez1-KO mice but also had increased anxiety and deficits in PPI (198, 199). A GSK-3α KO mouse model actually had facilitated PPI, reduced immobility in the FST, and reduced aggression, suggesting a protective effect against both schizophrenia and depression-like behaviors; however, these mice also had increased anxiety, reduced locomotion, and deficits in fear memory (200). Mice with diminished serine racemase (Srr) activity were found to have deficits in sociability and PPI (201). Kalirin (Kalrn) KO mice not only showed a similar phenotype to the Srr model but also showed increased anxiety, deficits in spatial learning and memory, and deficits in working memory (202). Given that deficits in sociability are seen in both depression and schizophrenia, the Srr and Kalrn mouse models could be interpreted as models for schizophrenia only.

Fez1 is involved in intracellular transport and has functions in neurodevelopment (172, 203). PDE4B and Srr are involved in cAMP and NMDA neurotransmission, respectively, and therefore affect diverse aspects of neuronal functioning (204, 205). GSK-3α is a serine–threonine kinase, and has been implicated in neurodevelopment, neurotransmitter function, neuroinflammation, and synaptic plasticity (206–209). Kalrn is a brain-specific guanine nucleotide exchange factor (GEF) that is a known regulator of spine morphogenesis (202). In addition to their link through DISC1, many of these molecules have their own links to schizophrenia and depression. Human genetic studies and expression studies have associated PDE4B, FEZ1, SRR, and KALRN with schizophrenia (177, 210–213). PDE4 and GSK-3 are associated with the action of antipsychotics and antidepressants (207, 214). Additionally, SRR metabolite d-serine is known to be beneficial in schizophrenia (215). These molecules regulate schizophrenia and depression-associated pathways downstream of DISC1 and further implicate neurodevelopment, synaptic processes such as spine regulation, and cAMP and NMDA signaling in schizophrenia and depression-associated behaviors.

### Reelin

Reelin (*RELN*) has been identified as a top candidate gene for schizophrenia in genetic association studies (216–219). Reelin levels were also shown to be decreased in schizophrenia and bipolar disorder (220–222), and altered with antipsychotic, antidepressant, and mood stabilizing medications (223). Reelin is a glycoprotein that is critical for development. The characteristically disorganized cortex of reeler mice demonstrates the importance of reelin in neuronal migration (224–226). Reelin is also important in synapse formation and plasticity, neuronal development, glutamatergic neurotransmission, and adult neurogenesis (225, 227–230). The intricacies of reelin signaling have been intensively studied and go beyond the scope of this review (231). Briefly, the effects of reelin on behavior and its connections to schizophrenia and depression may be realized through multiple pathways, from alterations in glutamate signaling and synapse regulation to widespread neurodevelopmental effects related to neuronal migration. Further work is needed to understand the specific contributions of these different pathways in mediating the effects of reelin on behavior, as well as their relationship to specific neuropsychiatric disorders such as schizophrenia and depression.

Reelin-deficient “reeler” mice are not suitable for behavioral testing due to disruptions in motor activity (225). However, heterozygous reeler mice have been used as a model for schizophrenia, although their validity in this context has been questioned (232). Some studies found that heterozygous reeler mice have cognitive deficits in operant conditioning and executive function (233–235), deficits in PPI, LI, and fear conditioning (236–238), male-specific hyperactivity in the MK-801-ILAT (239), and anxiety-like behavior in the EPM and OFT (237, 240). Others groups found no significant effects in these mice (232, 235, 241). Heterozygous reeler mice were also found to have altered LTP, and brain region-specific alterations in NMDA receptor subunit levels and ratios (233, 239, 242). Teixeira et al. found that reelin overexpressing mice had normal behavior under basal conditions; however, the mice had reduced immobility time in the FST after chronic corticosterone treatment, and reduced cocaine sensitization in the cocaine-ILAT (243). Furthermore, reelin overexpression prevented ketamine-induced PPI deficits. This group found no significant differences in heterozygous reelin-deficient mice, adding to the controversial findings associated with this model. Reelin models demonstrate the difficulties that can be encountered when attempting to replicate behavioral effects across different labs. Minute differences in environmental and test conditions can have consequences on behavior. Nevertheless, there are multiple lines of evidence supporting a role for reelin in neuropsychiatric disorders.

### Maternal immune activation

Maternal infection during pregnancy has consistently been associated with increased schizophrenia risk (244, 245). Maternal immune activation (MIA) was shown to affect DNA methylation (246). Hence, epigenetic changes caused by immune challenge during critical periods of development may perturb important schizophrenia-related pathways, which interact with underlying genetic susceptibility and lead to the development of symptoms. Interestingly, MIA has not been associated with depression in humans. A recent study of over 6,000 subjects failed to find an association between prenatal viral infection and the development of non-psychotic depression (247). Nevertheless, animal models have demonstrated behaviors relevant to both schizophrenia and depression.

Multiple rodent models have been used to examine the effects of prenatal immune challenge, primarily in relation to schizophrenia (248). MIA models have been less thoroughly explored for endophenotypes of depression. Nevertheless, certain models have shown phenotypes relevant for both schizophrenia and depression. Maternal viral infection modeled by challenge with polyriboinosinic–polyribocytidilic acid (poly I:C) caused: schizophrenia-like deficits in PPI, LI, and psychostimulant hypersensitivity in adult, but not adolescent animals, abnormal hippocampal–prefrontal synchrony (an electrophysiological endophenotype for schizophrenia), and changes in DA metabolism and receptor binding (249–263). Adult poly I:C exposed mice had impairments in recognition memory that were rescued with clozapine, but not haloperidol, deficits in spatial working learning and memory, and increased anxiety-like behavior (250, 254–256, 264, 265). Reversal learning was either impaired or improved depending on the timing of poly I:C exposure (264). The mice also showed anhedonia in the SPT; although only offspring of mothers that lost weight as a result of poly I:C injection displayed this effect (266). Additionally, MIA has been used in conjunction with genetic models to study gene × environment (G × E) interactions. For example, poly I:C MIA exacerbated the schizophrenia-like phenotype in Disc1-L100P heterozygotes (Table 1) (195). Interestingly, poly I:C MIA at embryonic day 9 in DN-DISC1 tg mice caused the development of anxiety-like and depression-like behaviors that were not seen in untreated DN-DISC1 tg mice (194). These changes were associated with enlarged ventricles, reduced hippocampal serotonin (5-HT), and reduced reactivity in the hypothalamic–pituitary–adrenal (HPA) axis.

The aberrant neuroanatomy and DA signaling seen in MIA models has long been associated with schizophrenia (267, 268). While MIA has not yet been associated with depression in humans, MIA interacted with DISC1 mouse models to generate depression-like behaviors. It may be that MIA is linked specifically to comorbid depression, but does not affect the development of major depressive disorder on its own. The effects of MIA on gene expression, DA and 5-HT signaling, neuroanatomy, and the HPA axis may interact with other genetic risk factors such as disruptions in DISC1, which leads to the development of schizophrenia and depression comorbidity. Future research is needed to explore the effects of MIA in conjunction with genetic risk factors on comorbid depression.

### Social isolation stress

Increased feelings of loneliness and social isolation, and decreased family and social support were associated with an increased risk depression and suicidality in schizophrenia patients (269). This is consistent with the long-standing hypothesis that environmental stressors such as social isolation can trigger depression in genetically susceptible individuals (270–272). The stress associated with social isolation may therefore be a factor in triggering comorbid depression in schizophrenia patients.

Social isolation is known to cause a number of behavioral changes related to neuropsychiatric disorders (273). Isolation during a critical period of post-natal development after weaning in rats was shown to cause hyperactivity in the OFT, schizophrenia-like deficits in PPI, but not LI, increased social interaction and aggression, and increased anxiety-like behavior (274–284). Isolation-reared rats showed increased responses to rewarding stimuli, including increases in sucrose and ethanol preference, operant responding for ethanol, and initiation of drug self-administration (285–291). Additionally, social isolation was shown to cause cognitive deficits in executive function, reversal learning, and spatial learning (291–296); although, spatial learning deficits were not universally seen, with some groups seeing no change and others seeing improvements (293, 297, 298). Isolation stress has also been used in conjunction with genetic mouse models. Social isolation in DN-DISC1-Tg-PrP mice induced schizophrenia-like deficits in PPI and depression-like behavior in the FST that were not seen in the socially isolated wild type mice or DN-DISC1-Tg-PrP control groups (Table 2) (196). These changes were linked to glucocorticoid and epigenetic control of genes related to DA signaling.

Social isolation causes brain region-specific alterations in DA and 5-HT activity in rodents; for example, heightened dopaminergic activity in the nucleus accumbens and ventral striatum, but reduced DA function in the PFC (273). Isolation was also shown to cause alterations in the expression and localization of glutamate receptor subunits, decrease numbers of GABAergic interneurons in the hippocampus, and decrease hippocampal brain-derived neurotrophic factor (BDNF) (50, 299–302). Additionally, isolation affected dendritic spine density and morphology in the PFC, striatum, and hippocampus (303–306). Post-weaning social isolation may therefore influence the development of schizophrenia and depression-related behavior via epigenetic changes – possibly through the HPA axis – that cause alterations in neurosignaling. These alterations may also interact with genetic risk factors such as DISC1, resulting in the development of symptoms. Isolation stress demonstrates that environmental input can induce schizophrenia and depression-related behavioral deficits in animals and is an excellent tool to use in conjunction with genetic models to test G × E interactions.

### Honorable mentions

This review has focused on animal models that display both schizophrenia and depression-relevant endophenotypes. However, due to the large amount of overlap between risk factors for schizophrenia and depression, numerous genes show convergent evidence from human data for a shared association between these disorders. Animal models of these genes do not necessarily display endophenotypes for both schizophrenia and depression. Nevertheless, below are some of the genes relevant to both disorders despite less phenotypic relevance to comorbidity than the previous animal models.

#### Neuregulin 1

An association between neuregulin 1 (NRG1) and schizophrenia is strongly supported by human genetic studies (307–313). NRG1 SNPs have also been associated with depression and bipolar disorder (314–318). NRG1 is a member of a family of epidermal growth factor-like proteins, which interact with the ErbB family of receptor tyrosine kinases to play a role in neurodevelopment, neuronal migration, Schwann cell growth, and brain activity homeostasis (319, 320).

While homozygous mice are embryonic lethal, heterozygous KO of NRG1, and its various isoforms display multiple schizophrenia-related behavioral deficits, including impaired PPI and LI, hyperactivity in the OFT, deficits in fear conditioning, impaired working memory, and/or abnormal social behavior (321–330). Overexpression of NRG1 also results in deficits in PPI, hyperactivity, and impairments in working memory, contextual fear conditioning, and social interaction (331–335). Additionally, heterozygous NRG1-KO mice show an increased sensitivity to the cannabinoid delta9-tetrahydrocannabinol (THC) and altered behavior in response to chronic social defeat stress (336, 337). Notably, social defeat caused impaired working memory and decreased aggression in NRG1 mice, but reduced deficits in sucrose preference relative to wild type mice (337). The relevance of NRG1 models to depression is weaker. While cognitive and social deficits and the altered response to psychosocial stress overlap as endophenotypes for schizophrenia and depression, there is much less research examining NRG1-KO mice in the specific context of depression. Future research and improvements in depression-related endophenotypes will reveal the utility of NRG1 models in studying comorbidity directly. Nevertheless, these models remain as excellent tools for understanding a pathway that shows convergent evidence for both depression and schizophrenia associations in humans.

#### Catechol-*O*-methyltransferase

Catechol-*O*-methyltransferase (COMT) is a primary DA metabolizing enzyme in the PFC and amygdala (338). COMT has been of particular interest in human studies because of a functional polymorphism (Val158Met), which is associated with a three to fourfold reduction in enzymatic activity and increased synaptic DA activity (339). While the COMT gene is located in a region associated with high schizophrenia risk (22q11), associations between the gene itself and schizophrenia have been inconsistent (340, 341). Likewise, COMT genetic variation does not appear to be associated with depression diagnosis or severity, and there is conflicting evidence for an association with response to antidepressants (342, 343). Nevertheless, COMT – particularly the Val158Met allele – is associated with a number of human endophenotypes, which are important in schizophrenia and depression, including PFC-mediated cognition, variations in brain structure, and anxiety traits (344–350). COMT has also been associated with violent behavior in schizophrenia patients (351, 352). COMT is therefore a potentially important player in linking certain cognitive and neuroanatomical symptom domains of depression and schizophrenia to the DA system.

COMT-KO mice exhibited an attenuated response to inhibition of DA transporter (DAT) and amphetamine in the ILAT, female-specific increases in anxiety-like behavior, male-specific increases in aggression, altered exploration and habituation in the OFT, and increased vulnerability to the disruptive effects of THC (353–358). Male COMT-KO mice displayed mild improvements in spatial and working memory (359, 360). Additionally, pharmacological inhibition of COMT improved attentional set-shifting performance in rats (361). Transgenic mice expressing the human COMT Val variant had impairments in attentional set-shifting, recognition memory, and working memory (360). Also, pain sensitivity and stress reactivity were decreased in transgenic mice and increased in COMT-KO mice. While COMT mice lack classical depression and schizophrenia-related endophenotypes, the models demonstrate relevance for cognitive function, which is an important aspect of both disorders. These models are therefore excellent tools for examining the role of DA function in cognition.

#### Brain-derived neurotrophic factor

Brain-derived neurotrophic factor is the most well studied and characterized neurotrophin in the CNS; we can only briefly touch upon the considerable literature here (362–365). BDNF and its high affinity tropomyosin-related kinase B (TrkB) receptor are involved in many important neuronal processes, including neurodevelopment, axon targeting, neuronal growth and survival, and synaptic plasticity (362). Evidence for altered brain and serum BDNF levels in schizophrenia is controversial, with studies finding both increased and decreased levels in various brain regions (366–372). However, the BDNF Val66Met polymorphism has been associated with increased schizophrenia risk (373). Furthermore, the BDNF Val66Met allele was shown to interact with childhood trauma to decrease blood BDNF mRNA levels and hippocampal subfield volumes in schizophrenia and bipolar disorder patients, suggesting a G × E interaction that may have consequences on brain development and function in psychosis (374). Decreased BDNF levels have been consistently reported in depression, particularly in suicidal patients (375–382). Indeed, a role for BDNF in the pathophysiology and treatment of depression and schizophrenia is strongly supported.

Many mouse lines have been developed with various mutations in BDNF. Homozygous mice possessing a BDNF null mutation are not viable. However, heterozygotes display many relevant phenotypes, including hyperactivity, hyperphagia causing excess weight gain, potentiated response to amphetamine in the ILAT, aggression, impaired contextual fear conditioning, extinction learning deficits, and sex-specific vulnerability to the behavioral effects of THC and corticosterone (383–392). Heterozygous BDNF-KO mice showed baseline PPI deficits only in paradigms involving chronic injection, suggesting that this may be a stress-induced effect (386, 391, 392). Supporting the susceptibility of PPI to environmental factors in heterozygous BDNF-KO mice is the finding that cannabinoid and methamphetamine treatment in young-adult mice caused sex-specific changes in PPI response to acute cannabinoid and amphetamine challenge, respectively, in adult heterozygous mice relative to both wild type and untreated heterozygotes (386, 392). Depression-related endophenotypes such as learned helplessness, anhedonia, and vulnerability to stress were not seen in heterozygous BDNF-KO mice (393–395). However, the response to amine-based antidepressants is attenuated in this model (396). Conditional fetal, post-natal, hippocampal, and forebrain-inducible BDNF-KO mice displayed depression-like behaviors in certain tests (397–399). Forebrain-specific BDNF-KO mice displayed learning and memory deficits (400). Additionally, a mouse model of the human Val66Met allele displayed increased aggression, anxiety, and deficits in contextual fear conditioning (401). BDNF overexpressing mice showed improved learning and memory in the MWM and reduced immobility in the FST (402, 403).

Mice lacking BDNF receptor TrkB in the brain demonstrated a similar phenotype, displaying hyperactivity, and increased impulsivity in the NORT, but not depression-like or anxiety-like behaviors in the FST or EPM (404). Conversely, mice overexpressing TrkB show improvements in spatial learning and memory, contextual fear conditioning, and reduced anxiety in the EPM (405). Finally, the importance of environmental factors to BDNF is supported by the finding that maternal separation and adolescent/young-adult corticosterone treatment caused sex and brain region-specific changes in BDNF and TrkB function coupled with male-specific deficits in working memory and female-specific anhedonia in the SPT (406).

While related endophenotypes of depression and schizophrenia are seen in various BDNF models, they are not seen simultaneously in the same model. More research on BDNF models in the context of both schizophrenia and depression is needed. Nevertheless, various disruptions in the BDNF pathway do lead to both schizophrenia and depression-related behavioral deficits. This suggests a role for BDNF in a shared pathway between the two disorders. Parsing the differences that lead to specific disruptions in behavior will greatly aid in elucidating the contributions of the BDNF pathway to depression and schizophrenia.

## Bringing the Picture Together

**Figure 1:**
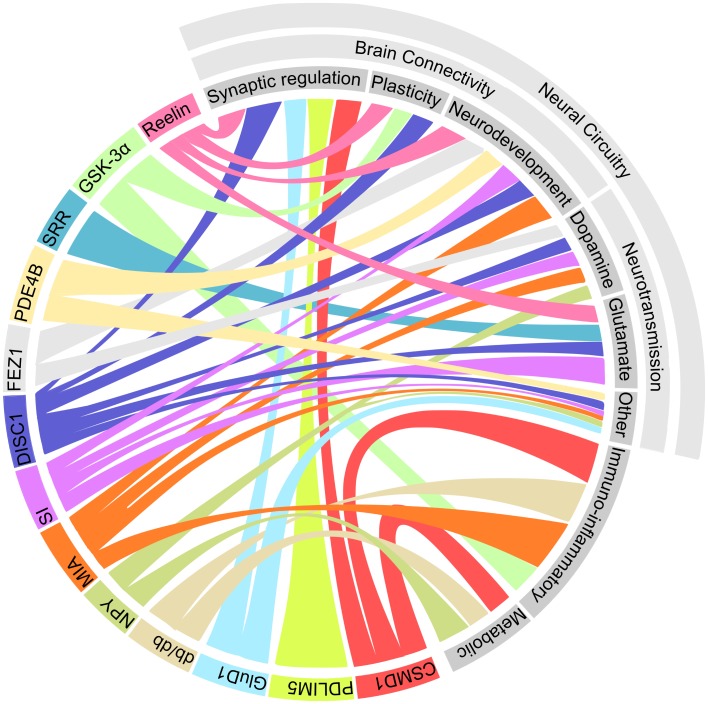
**Multiple shared pathways between rodent models which display both schizophrenia and depression-related phenotypes**. This diagram illustrates the connections between each of the models (represented in color on the bottom right) and the biological processes which potentially underlie the observed phenotypes (represented in gray at the top left). *Abbreviations*: CSMD1, CUB and SUSHI multiple domains 1; DISC1, disrupted in schizophrenia 1; FEZ1, fasciculation and elongation protein zeta 1; GluD1, glutamate receptor delta 1; GSK-3α, glycogen synthase kinase 3α; MIA, maternal immune activation; NPY, neuropeptide Y; PDE4B, phosphodiesterase 4B; PDLIM5, PDZ and LIM domain 5; SRR, serine racemase; SI, social isolation.

The emerging picture of the genetic architecture of schizophrenia is revealing that hundreds of genes with small effect sizes influence the disorder (407). The genetic picture of depression is far less clear, with heritability estimates predicting a much greater contribution of environmental effects than in schizophrenia (408, 409). Hence, it is not surprising that the array of factors that influence depression and schizophrenia-related phenotypes in rodent models is diverse. However, a number of common elements between these models are becoming evident. The emerging pathways that are shared between these models are represented in Figure 1.

### Neurotransmission: The familiar suspects

Most current antipsychotics and antidepressants affect neurotransmitters in the synapse. A number of the models mentioned have demonstrated links to various neurotransmitter systems. NPY, DISC1, and Fez1 pathways interact with the DA system (157, 158, 188, 197, 410, 411). Altered DA and 5-HT activity was seen after MIA and social isolation. Furthermore, the increased sensitivity to psychostimulants seen in a number of the aforementioned models is thought to be related to DA activity (412). A role for glutamate is also implicated in many of these models. Srr, DISC1, and reelin are all involved in glutamatergic signaling (205, 413, 414). Social isolation also affected NMDA receptor localization (299). Many antipsychotics have been used in the treatment of depressive disorders (415). It is possible that the shared involvement of certain neurotransmitter systems in schizophrenia and depression underlies both the increased risk of comorbid depression in schizophrenia and the antidepressant activity of these antipsychotics.

Involvement of monoamine systems in schizophrenia and depression is by no means a new hypothesis (267, 416). DA, in particular, is strongly implicated in schizophrenia. Furthermore, it is easy to see how the mesocortical and mesolimbic DA reward circuits could be involved in anhedonia and amotivational states associated with both depression and schizophrenia. While these models provide additional support for this hypothesis, a number of questions remain regarding the role of monoamine systems in generating the phenotypes seen in these models. DISC1, NPY, and Fez1 are all involved in multiple pathways. Future experiments are needed to determine if the DA system alone is necessary and sufficient to account for specific observed phenotypes, or if it is peripheral or supplementary to the development of certain schizophrenia and depression-related endophenotypes. Likewise, MIA and social isolation affect more than just neurotransmitter systems. Some work has been done to uncover the molecular pathways by which these environmental factors cause perturbations in neurotransmitter systems; for example, social isolation caused DNA methylation of the promoter region of tyrosine hydroxylase in the ventral tegmental area of DN-DISC1-Tg-PrP mice. More similar studies are needed to uncover the complex G × E interactions which lead to altered neurotransmission in these models.

There is mounting evidence that glutamate plays a major role in psychiatric illness. The link between glutamate and schizophrenia was first proposed based on the observation that NMDA receptor antagonists phencyclidine (PCP) and ketamine can induce schizophrenia-like symptoms in healthy individuals (417–419). The hypothesis has since gained supporting evidence from human genetic and imaging studies, as well as animal models of NMDA receptor hypofunction (419–421). A relationship between the glutamate system and depression is suggested by the rapid and long-lasting antidepressant effects of ketamine (422). Early evidence is showing that compounds targeting the glutamate system may have efficacy in treating positive, negative, and cognitive symptoms of schizophrenia (423). The efficacy of these compounds in treating negative symptoms such as anhedonia and social withdrawal, which overlap with depression, may indicate potential antidepressant activity. Hence, the glutamate system is of particular interest in treating comorbid depression in schizophrenia. The current animal models will be useful for investigating the efficacy of new compounds targeting the glutamate system in treating symptoms of schizophrenia and depression.

### Connectivity is the key

Disruptions in processes related to brain connectivity are a common theme among the many of the models outlined here. Almost all of the models mentioned demonstrate links to synaptic processes such as synapse formation, regulation, and plasticity. Pdlim5, GluD1, reelin, Disc1, Kalrn, and social isolation affect synaptic spine morphology and/or formation, and GSK-3, Disc1, and reelin affect synaptic plasticity (107, 121, 134, 135, 202, 209, 231, 424, 425). Dynamic changes in synaptic spine morphology and formation, both developmentally during the establishment of neuronal circuits and as the result of activity or experience-dependent remodeling of existing circuits, are thought to be intimately linked to cognitive development and function (426–429). Disruptions in neurodevelopment may also interfere with brain connectivity through the “miswiring” of neuronal circuits. Disc1 and reelin are both important for neuronal migration and the formation of cortex layers, and PDE4B is involved in axon guidance and dendritic growth (183, 186, 224). Miswiring of neuronal circuits, whether at the level of brain structure from abnormal neurodevelopment or from dysregulation at the level of the synapse, possibly cause maladaptive alterations in brain connectivity leading to altered stimulus processing and cognition. This may be a common mechanism underlying the cognitive and behavioral symptoms of both schizophrenia and depression.

Broad constructs such as connectivity, synaptic regulation, and plasticity are far too general to lead to significant advancement in the mechanistic understanding or treatment of neuropsychiatric illness (430). Furthermore, these mechanisms are implicated in a myriad of disorders in addition to depression and schizophrenia; for example, neuronal circuit dysfunction is also implicated in intellectual disability, ASD, and Alzheimer’s (431). Future research should examine the precise changes in specific neural circuitry and synaptic processes in these and forthcoming models of schizophrenia and depression. Models such as the Pdlim5-KO and Fez1-KO mice are of particular interest as they display endophenotypes of one disorder while being protective against endophenotypes of the other. Hence, these molecules may represent points in the shared pathway where schizophrenia and depression diverge. Examining these mechanisms could hint at the subtle changes that can cause the emergence of disparate symptoms in disorders with shared genetic susceptibility. Research correlating precise changes in neural circuitry and synapse function to specific disease-related endophenotypes in these animal models will be important in completing the picture linking genetic changes to pathophysiology and ultimately behavior.

### Immune and environmental factors

Dysregulation of the immune system, cytokines, and oxidative and nitrositive stress have been proposed as important factors in both schizophrenia and depression (432, 433). This hypothesis is supported by a myriad of evidence from the study of immuno-inflammatory markers in humans, genetic association studies, and animal models (434, 435). Immune and inflammatory processes were implicated in schizophrenia and depression comorbidity by a number of models, including Csmd1, db/db, GSK-3, and MIA (145, 208, 248). Furthermore, Csmd1 provides a link between the immune system and neuronal processes such as synaptic pruning (107). It has been proposed that schizophrenia is immunologically primed for the expression of depression (434). This is supported by the aforementioned models, particularly by findings such as the interaction between MIA and Disc1 in mice to illicit depression and anxiety-like behavior (194).

However, a number of key questions remain in understanding the role of immune and inflammatory processes. Future research should reveal the extent to which these processes are responsible for the observed phenotypes. MIA-induced locomotor changes could be rescued by maternal treatment with non-steroidal anti-inflammatory drugs or adolescent treatment with the COX-2 inhibitor celecoxib (436, 437). Similar investigations could be done using other models; for instance, using the db/db mice in which hippocampal inflammation is thought to be an important factor in the observed phenotype. Furthermore, determining the sensitivity of treatment with anti-inflammatory drugs at different time points will reveal if immune insult leads to permanent changes in brain structure and function. This will be important for developing new therapeutic strategies for schizophrenia and depression.

Models of MIA and social isolation stress demonstrate how environmental factors can cause broad changes in neurobiology and behavior. Heritability is estimated at 81% for schizophrenia and 37% for depression (408, 438). This means almost 1/5th of the estimated variance in liability for schizophrenia and 3/5ths for depression is due to non-genetic factors. Clearly, environmental factors play an important role in influencing brain function, and are modulated by both genetic and epigenetic factors (439). Several environmental stressors such as psychosocial stress, drug abuse, nutrition, and MIA influence schizophrenia and depression in humans, as well as related endophenotypes in mice (440, 441). Environmental factors have already been combined with Disc1 genetic models to explore the complex and synergistic G × E interactions, which trigger the development of pathological endophenotypes (194–196, 442, 443). Additionally, two-hit models have been used combining factors such as acute and chronic response to THC, psychosocial stress, and chronic unpredictable stress with models of NRG1, COMT, BDNF, and other genes (336, 337, 358, 444). Future research should apply this type of hybrid G × E approach to other combinations of genes and environmental factors to improve our understanding of how genes modulate sensitivity to environmental stressors and lead to mental illness. Additionally, correlating these effects with changes in the epigenome will improve our understanding of the sequence of molecular events which lead to the emergence of symptoms in depression and schizophrenia.

### Metabolic syndrome: Culprit, accomplice, or bystander?

Patients with both schizophrenia and depression are at an increased risk for components of metabolic syndrome, including obesity, hypertension, atherogenic dyslipidemia, hyperglycemia, and diabetes (445–447). Metabolic syndrome in schizophrenia patients can be partially explained as a side-effect of antipsychotic medications (448). Nevertheless, a common mechanism between these conditions is hinted at by the db/db, Csmd1, and NPY models (108, 145, 161–163). Research linking metabolic syndrome and mental illness is still relatively new. Systemic inflammation and immune activation are features of schizophrenia, depression, and metabolic syndrome (434, 447). Hence, immune dysregulation could be a causal factor in all three disorders. This is supported by fact that knocking out the immune molecule Csmd1 led to the development of glucose tolerance as well as schizophrenia and depression endophenotypes in mice (108). Alternatively, disruptions in systems such as leptin and NPY, which are involved in hunger and satiety, may cause schizophrenia and depression, while simultaneously predisposing patients of these disorders to behavioral risk factors for metabolic syndrome such as poor diet and sedentary lifestyle.

Whether metabolic syndrome is a causal factor, a consequence, or simply a marker of mental illness is subject to controversy (449). Future studies should determine if treating metabolic deficits in models such as the Csmd1-KO and db/db mice rescues schizophrenia and depression-related endophenotypes. This would help to elucidate the causal status of metabolic conditions in schizophrenia and depression endophenotypes. A detailed discussion of the other links between depression, schizophrenia, and metabolic disorder goes beyond the scope of this review (446, 447). More research is needed to reveal the relationship between these disorders.

## Whole Picture and Future Directions

Ultimately, all of the factors mentioned here are intimately interconnected. Dopaminergic, glutamatergic, and GABAergic neurotransmitter systems interact as neural circuits and are influenced by inputs from multiple other systems (267, 420, 450). Synaptic processes are regulated by neurotransmitters and immune molecules, and in turn affect neurotransmission (451–453). Finally, neurodevelopmental processes wire the machinery necessary for all this to occur, and are influenced by each of these factors (454, 455). Future research should then focus on differentiating precise mechanisms and their relationships to these highly integrated systems. Advancing technologies such as optogenetics and light sheet microscopy should aid in deciphering the roles of specific neural circuitry (456). Neural circuits can be further interrogated through the use of genetic approaches in simple model organisms such as *Drosophila* larvae and *Caenorhabditis elegans* (457, 458). Conversely, applying genetic techniques to more complex organisms such as the rat will allow for the assessment of more sophisticated cognitive and social behaviors (174).

While this review specifically focused on schizophrenia and depression, Table 1 emphasizes that the behavioral endophenotypes used in these studies are linked to multiple disorders. Furthermore, many of the genes targeted in these models have multiple associations; for example, DISC1 is associated with bipolar disorder, major depression, social anhedonia, chronic fatigue syndrome, anxiety, neuroticism, emotional stability, schizophrenia, schizoaffective disorder, and ASD (179, 459, 460). Given this level of complexity, it is unrealistic to assume that disorders such as schizophrenia and depression can be sufficiently approximated and recognized in rodent models, especially considering the controversy in psychiatric nosology itself regarding the definition of discrete boundaries between disorders (461). Hence, it may be useful to focus on correlating specific molecular pathways with certain endophenotypes rather than attempting to interpret models as holistic representations of mental illness. Considering the multidimensional nature of both depression and schizophrenia, understanding causal mechanisms as they relate to certain dimensions of symptomology reflected in specific endophenotypes may provide insight into the genetic origins of the heterogeneous and multidimensional nature of these disorders.

Lastly, if we desire to use animal models for translational research in drug discovery, we will need models with greater etiological validity. Schizophrenia and depression are highly polygenetic, likely resulting from the contribution of multiple low-effect genetic risk factors combined with environmental stressors (407, 409). Less severe and more specific genetic manipulations such as point mutation models, genetic hypomorphs, and RNAi gene knockdown methods cause more subtle changes that better mimic the types of genetic factors seen in human populations (174). Furthermore, multi-hit models, which combine multiple mutations and environmental factors, more closely model the polygenetic nature of these disorders, and allow for the interrogation of both gene × gene and G × E interactions. New methods such as the CRISPR/Cas system allow for the one step generation of multiple mutations to greatly accelerate the development of such models (462).

## Conclusion

What has emerged so far from the study of animal models exhibiting both depression and schizophrenia-related phenotypes is a number of broadly defined mechanisms, which may underlie a shared pathophysiology between the two disorders. Given the complex, heterogeneous nature of these disorders, it is likely that neurotransmission, brain connectivity, immune, and environmental factors all contribute to their pathophysiology. As more models are discovered, the emerging picture of the shared pathophysiological mechanisms between schizophrenia and depression will become increasingly coherent. Interrogating precise molecular and neural substrates as they relate to specific endophenotypes, and carefully examining gene × gene and G × E interactions will contribute to a better understanding of the neurobiological mechanisms of comorbidity in mental illness. This understanding will inform future efforts in developing treatments for neuropsychiatric comorbidity.

## Conflict of Interest Statement

The authors declare that the research was conducted in the absence of any commercial or financial relationships that could be construed as a potential conflict of interest.
